# Genetic inhibition of the atypical kinase Wee1 selectively drives apoptosis of p53 inactive tumor cells

**DOI:** 10.1186/1471-2407-14-430

**Published:** 2014-06-13

**Authors:** William N Pappano, Qian Zhang, Lora A Tucker, Chris Tse, Jieyi Wang

**Affiliations:** 1 Discovery Research, AbbVie Inc., 1 N. Waukegan Road, North Chicago, IL 60064, USA

**Keywords:** Wee1, p53, Apoptosis, CDK1, CDK2, DNA damage

## Abstract

**Background:**

Tumorigenesis is the result of genomic or epigenomic insults and subsequent loss of the proper mechanisms to respond to these alterations leading to unscheduled growth. Tumors arising from these mutations often have altered cell cycles that offer proliferative advantages and lead to the accumulation of additional mutations that can lead to more aggressive phenotypes. Nevertheless, tumor cells must still adhere to the basic tenets of the cell cycle program to ensure their survival by DNA duplication, chromosomal segregation and cytokinesis. The atypical tyrosine kinase Wee1 plays a key role in regulating the cell cycle at the DNA synthesis and mitotic checkpoints via phosphorylation and subsequent inactivation of cyclin-dependent kinases (CDKs) in both healthy and tumorigenic cells.

**Methods:**

To assess the role of Wee1 in tumor cell proliferation we performed small interfering RNA (siRNA) experiments in a panel of diverse cell lines derived from various tissue origins. We also tested the hypothesis that any potential effects would be as a result of the kinase activity of Wee1 by siRNA rescue studies with wild-type or kinase-dead versions of Wee1.

**Results:**

We find that, in general, cells with wild-type p53 activity are not susceptible to loss of Wee1 protein via siRNA. However, Wee1 siRNA treatment in tumor cells with an inherent loss of p53 activity results in a deregulated cell cycle that causes simultaneous DNA synthesis and premature mitosis and that these effects are kinase dependent. These cumulative effects lead to potent inhibition of cellular proliferation and ultimately caspase-dependent apoptosis in the absence of co-treatment with cytotoxic agents.

**Conclusions:**

These results suggest that, while Wee1 acts as a tumor suppressor in the context of normal cell growth and its functional loss can be compensated by p53-dependent DNA damage repairing mechanisms, specific inhibition of Wee1 has deleterious effects on the proliferation and survival of p53 inactive tumors. In total, targeting the atypical kinase Wee1 with an siRNA-based therapeutic or a selective ATP competitive small molecule inhibitor would be a feasible approach to targeting p53 inactive tumors in the clinic.

## Background

Proper maintenance of the cell cycle is essential for the development and homeostasis of all living organisms. Neoplasms arising within these organisms also rely on a coordinated cell cycle to facilitate their rapid growth rate in response to external sources of nutrients and signaling stimuli. Progression through the cell cycle in both naïve and tumor tissue is monitored at checkpoints that sense possible defects in DNA synthesis and chromosomal segregation. Activation of these checkpoints results in cell cycle arrest and allows cells to rectify any negative perturbations that may be transmitted to their resulting daughter cells. Tumor cells with defective checkpoints rely more heavily on other checkpoints within the cell cycle to ensure their survival. Thus, deregulation of cell cycle control can have catastrophic results and therefore has led to a concerted effort towards generation of novel therapeutic agents targeting the cell cycle in tumors [1].

Transition through the cell cycle is dependent on the cyclin-dependent kinase (CDK) family of regulatory proteins. CDK activity is tightly monitored through several complex mechanisms including modulation of CDK stability by binding partner cyclins and CDK inhibitors [2,3]. However, only a subset of CDK-cyclin complexes are directly involved in progression of the cell cycle through these respective checkpoints. The active CDK2-Cyclin E complex is essential to drive the G1/S transition of the cell cycle after the restriction checkpoint and the CDK1-Cyclin B complex (also known as the M-phase-promoting-factor) is the master regulator that initiates the G2/M transition after the mitotic checkpoint [4]. In addition to their association with cyclins and CDK inhibitors, CDK1 and CDK2 activity are modulated both negatively and positively by phosphorylation and de-phosphorylation events [4]. Wee1 is an atypical tyrosine kinase that most closely resembles serine/threonine kinases in both sequence and structure [5] and acts directly upon CDK1 and CDK2. Wee1 phosphorylation of tyrosine 15 (Y15) of CDK1 and CDK2 results in CDK inactivation and inhibition of S-phase and mitotic entry [6,7]. Wee1 kinase activity therefore serves as a master regulator of cell cycle checkpoints by inactivating the CDK1-Cyclin B and CDK2-Cyclin E complexes until it can be assured that genomic integrity will be maintained and that the appropriate genetic information will be passed on to daughter cells.

Previous reports of Wee1 inhibition by small molecule kinase inhibitors and siRNA demonstrate that loss of Wee1 activity sensitizes p53 inactive cells to DNA damaging agents and radiosensitization [8-12]. We hypothesized that loss of Wee1 in the absence of cytotoxics should be able to affect tumor cell proliferation because all metazoan cells, including cancer cells, rely on at least partially functioning checkpoints to insure their survival. To elucidate the roles of Wee1 in cancer cell cycle progression we have utilized siRNA knockdown and rescue. We find that loss of Wee1 kinase activity results in dramatic cell cycle events including simultaneous mitosis and DNA synthesis that ultimately lead to apoptosis in a sub-set of p53 deficient cells. The anti-proliferative and apoptotic effects of Wee1 siRNA treatment can be circumvented by expression of a wild-type Wee1 rescue construct but not a kinase-defective version in affected target cells. This work provides new insights into the development of cancer therapeutics, suggesting that a small molecule inhibitor of Wee1 kinase should be efficacious against a large number of p53 inactive solid tumors as a single agent and provide a safe therapeutic window in the p53 wild-type tissues of patients.

## Methods

### Cell culture

All cell lines were obtained from ATCC (Manassas, VA, U.S.A.). All cell lines were grown according to manufacturer’s conditions in the presence of fetal bovine serum (Invitrogen, Carlsbad, CA, U.S.A.).

### siRNA oligos and transfection

All siRNAs were obtained from the ON-TARGET *plus* collection purchased from Dharmacon (Lafayette, CO, U.S.A). The sense strand sequences of the Wee1 siRNA oligos employed in the study are as follows: #5 5′-AAUAGAACAUCUCGACUUA-3′, #6 5′-AAUAUGAAGUCCCGGUAUA-3′, #7 5′-GAUCAUAUGCUUAUACAGA-3′, #8 5′-CGACAGACUCCUCAAGUGA-3′. The negative control siRNA oligos: NT1 and NT2 product numbers D-001810-01 and D-001810-02. The Ran oligo targeting sequence was 5′- AGAAGAAUCUUCAGUACUAUU-3′. Transfections of siRNA duplexes were performed using RNAiMAX reagent (Invitrogen).

### Cell proliferation and caspase assays

Viable cell numbers were measured using CellTiter-Glo reagent (Promega, Madison, WI, U.S.A.) or CyQuant (Invitrogen). Caspase-Glo 3/7 assay (Promega) was used to measure cellular caspase-3/7 activity.

### Antibodies and Western blot analysis

Antibodies used included mouse anti-Wee1, mouse anti-P53 (Santa Cruz Biotechnology, Santa Cruz, CA, U.S.A.), rabbit anti-phospho-CDK1 (Y15), rabbit anti-phospho-H2AX (S139), mouse anti-phospho-Histone H3 (S10), rabbit anti-Histone H3 (Cell Signaling Technology, Danvers, MA, U.S.A.), mouse anti-CDK1 (Millipore, Billerica, MA, U.S.A.), mouse anti-P21 (BD Biosciences, San Diego, CA, U.S.A.), and mouse anti-Actin (Sigma, St. Louis, MO, U.S.A.). Western blots were performed as previously described [13].

### DNA cloning and Engineered cell lines

The human Wee1 coding sequence was amplified using standard PCR protocols and cloned into the pLVX-puro lentiviral construct (Clontech, Mountain View, CA, U.S.A.). Kinase-altered Wee1 (K328R) and siRNA resistant constructs were introduced using the QuikChange site directed mutagenesis kit (Stratgene, Santa Clara, CA, U.S.A.). Cell lines were generated immediately after infection by mass selection in 2 μg/ml puromycin (Clontech).

### siRNA rescue experiment

Engineered NCI-H1299 cell lines were grown in DMEM containing 10% fetal bovine serum and 2 μg/ml puromycin. Cell lysate samples were collected 48 hr after transfection for Western blot analysis. Five days after siRNA transfection, microscopic cell images were taken and both floating and attached cells were then collected by trypsinization and counted for viable cell number using a Vi-CELL cell viability analyzer (Beckman Coulter, Brea, CA, U.S.A.).

### Time-lapse light microscopy movies

NCI-H1299 and A549 cells were plated and transfected 24 hours later with either control siRNA (NT1) or Wee1 siRNA (#8). Plates were placed in an incubator and images were captured every 30 minutes using IncuCyte™ High Definition (HD) Imaging (Essen Bioscience, Ann Arbor, MI, U.S.A). Movies were extracted for the indicated time frames (3 days of siRNA treatment) at a rate of 10 frames per second.

### Analysis of cell cycle by flow cytometry

At varied times after transfection with 5nM siRNA, adherent cells were trypsinized and combined with any floating cells present, then washed with cold PBS. Cells were then stained with 0.5 ml PBS containing 50 μg/ml propidium iodide (PI), 0.1 mg/ml RNase A, 0.1% BSA, and 0.1% Triton-X100 for 20 min and cell cycle distribution was analyzed using a BD LSR-II flow cytometer (BD Biosciences, San Jose, CA, U.S.A.).

### Double thymidine block

H1299 cells were treated with 2 mM thymidine (Sigma) and blocked for 20 hrs. Cells were then released by washing with PBS and feeding with regular culture medium. Four hours after the first release, cells were transfected with 5nM siRNA and incubated for another 4.5 hrs before the second treatment of 2 mM thymidine. Cells were blocked with thymidine for another 14.5 hrs and released again into regular medium. At the second release (t = 0), cells should be synchronized at G1/S phase boundary of the cell cycle. Cell samples were collected at varied time points for cell cycle and Western blot analysis as described above.

### Click-iT EdU flow cytometry assay

NCI-H1299 cells were plated in 6-well plate and transfected with 5 nM siRNA as described above. After 24 or 48 hr incubation, cells were labeled with 10 μM EdU (Invitrogen) for 30 min. Both floating and attached cells were then collected for fixation, Click-iT reaction, and cell cycle staining following manufacturer’s protocol. Cell samples were analyzed for DNA content and EdU level using BD LSR-II flow cytometer.

## Results

### Wee1 siRNA treatment results in potent inhibition of proliferation in a subset of human solid tumor cell lines

**Figure 1:**
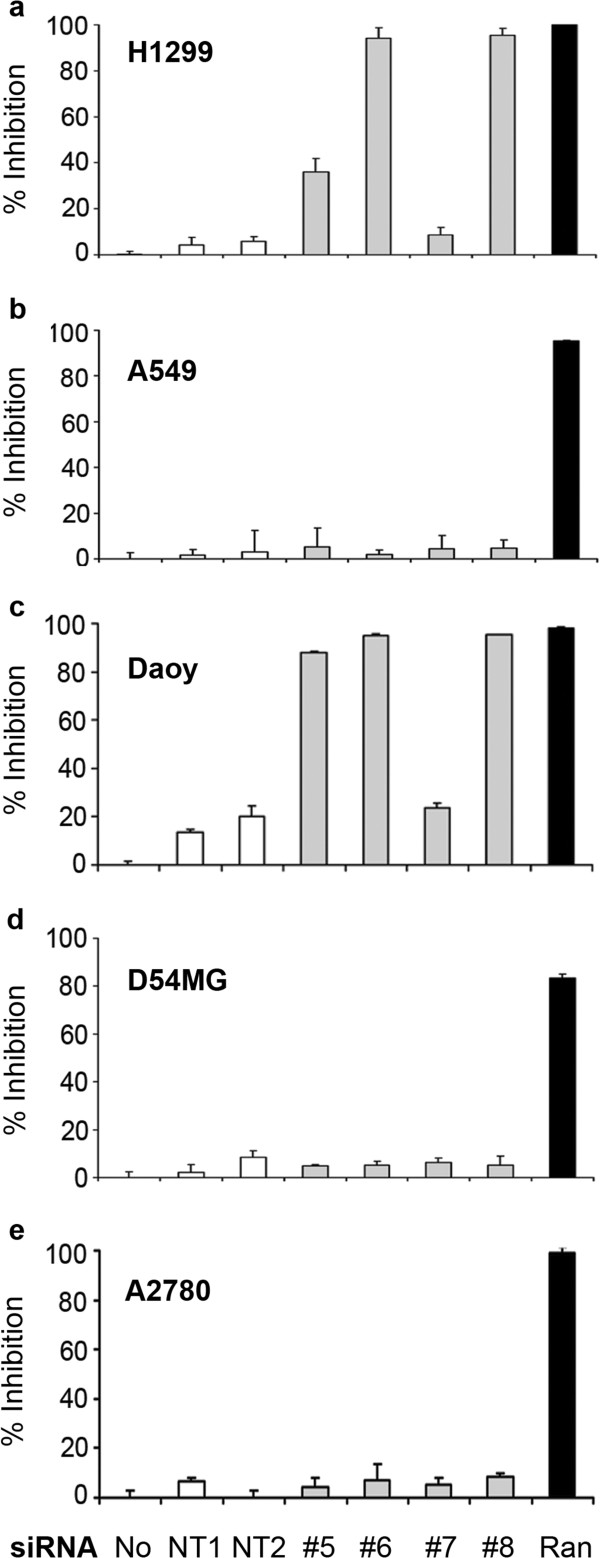
**Wee1 knockdown results in potent inhibition of proliferation in a subset of human tumor cell lines. (a-e)** NCI-H1299, A549, Daoy, D54MG and A2780 cell lines were subjected to 5 nM siRNA treatment for five days following plating and end-point analysis was performed using CellTiter-Glo . Controls included no siRNA (No), non-targeting siRNAs (NT1 and NT2) as negative controls and Ran siRNA (Ran) as a positive control for transfection efficiency. Four individual Wee1 siRNAs were used (#5-#8). Percent inhibition was calculated using the negative control (No siRNA) as 0% inhibition and the positive control (Ran siRNA) as 100% inhibition to control for relative transfection in each individual cell line. Results represent triplicate counts from a representative experiment with error bars representing s.e.m.

Previous published reports on Wee1 inhibition by small molecule intervention or siRNA knockdown provide conflicting results regarding whether the loss of Wee1 kinase activity can result in the inhibition of growth in tumor cell lines [8,10-12,14]. To test the hypothesis that ablation of Wee1 in the absence of cytotoxics would have effects on cellular health we performed siRNA experiments on a panel of human cell lines derived from solid tumors of diverse origin. Four distinct siRNAs targeting Wee1 as well as negative (non-targeting siRNA #1 and #2) and positive (Ran, [15]) controls were added to cells for the duration of the experiments. The negative controls ensure that siRNA transfection has no deleterious effects on cell growth while the positive control provides a reference of maximum cell killing possible and ensures transfection efficiency. The resulting phenotypes of Wee1 siRNA resulted in dramatic inhibition of cellular proliferation as measured by MTS reagent in H1299 non-small cell lung (NSCLC) and Daoy glioma cell lines but did not affect growth of A549 NSCLC, D54MG glioma or A2780 ovarian tumor lines (Figure 1). Similar results were obtained using DNA content (CyQuant assay) as a measure of proliferation (Additional file 1).

**Figure 2:**
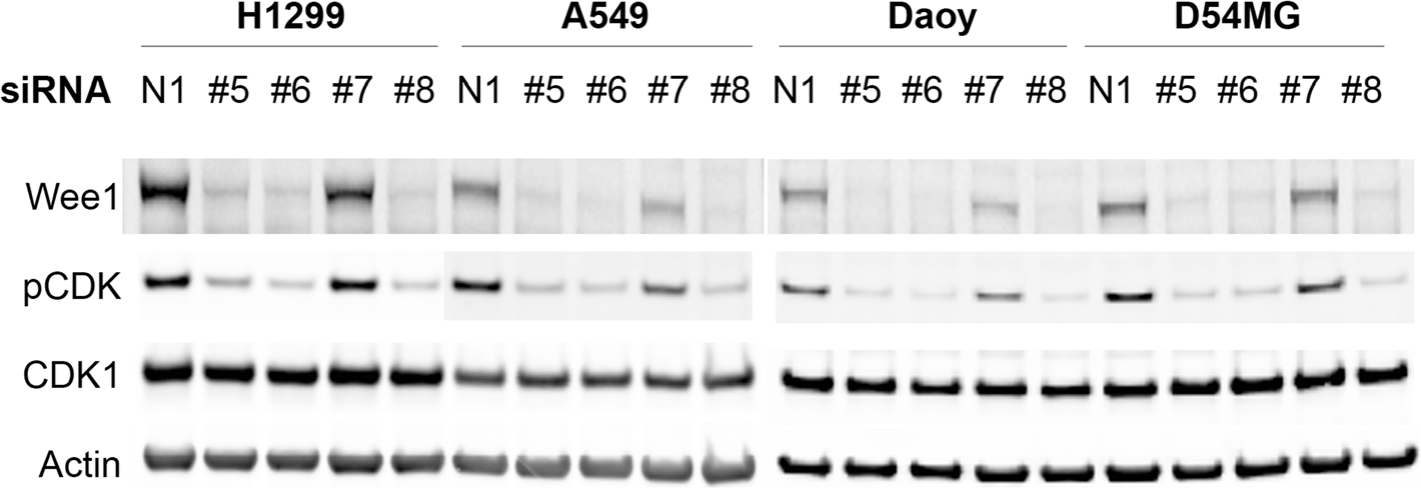
**Wee1 siRNA treatment produces efficient target knockdown in all cell lines assayed.** NCI-H1299, A549, Daoy and D54MG cells were subjected to 5 nM siRNA treatment for two days following plating. Non targeting siRNA (N1) and Wee1 siRNAs #5-#8 were all analyzed. Direct cell lysates were analyzed by Western blotting to ascertain loss of Wee1 protein levels as well as the effects on its direct target (pCDK). pCDK Western samples were spliced between H1299 and A549 to provide exposure times that result in similar levels of visible phosphorylation for all cell lines. Total CDK1 and Actin were blotted to control for total protein levels.

The striking differences between responding and non-responding tumor cell lines to Wee1 siRNA could not be easily explained by off-target effects because of the complete lack of efficacy in A549, D54MG and A2780 cells. However, one possible explanation for the mixed phenotypic effects of Wee1 siRNAs was differential loss of the Wee1 protein. To explore this possibility, we performed Western blot analysis of all five cell lines after siRNA transfection to assess the loss of Wee1 protein. We also examined pCDK which served as a biomarker readout for loss of Wee1 activity in these cells. siRNA treatment resulted in a roughly equivalent loss of Wee1 protein from effective siRNAs in all cell lines examined (Figure 2 and Additional file 2). Moreover, the loss of Wee1 kinase activity also appeared to be roughly equivalent in each of the tumor cell lines based on the levels of CDK Y15 phosphorylation (Figure 2 and Additional file 2). The siRNAs targeting Wee1 used in this study produced protein knock-down and downstream effects over a range of low nanomolar concentrations in all cell lines analyzed (data for Daoy cells shown in Additional file 3). It should be noted that the peptide-based pCDK monoclonal antibody that is used in this study and others cannot distinguish between CDK1, CDK2 and CDK5 phosphorylation as each of these kinases have identical peptides in the region surrounding the Y15 phosphorylation site. In total, these results indicate that differential loss of Wee1 protein or kinase activity amongst cell lines is not the cause of the dramatic differences seen in cellular phenotypes after Wee1 siRNA treatment. These data indicate that in a number of cancer cell lines Wee1 is absolutely essential for survival.

### Wee1 siRNA treatment is on-target and its effects are kinase activity dependent

**Figure 3:**
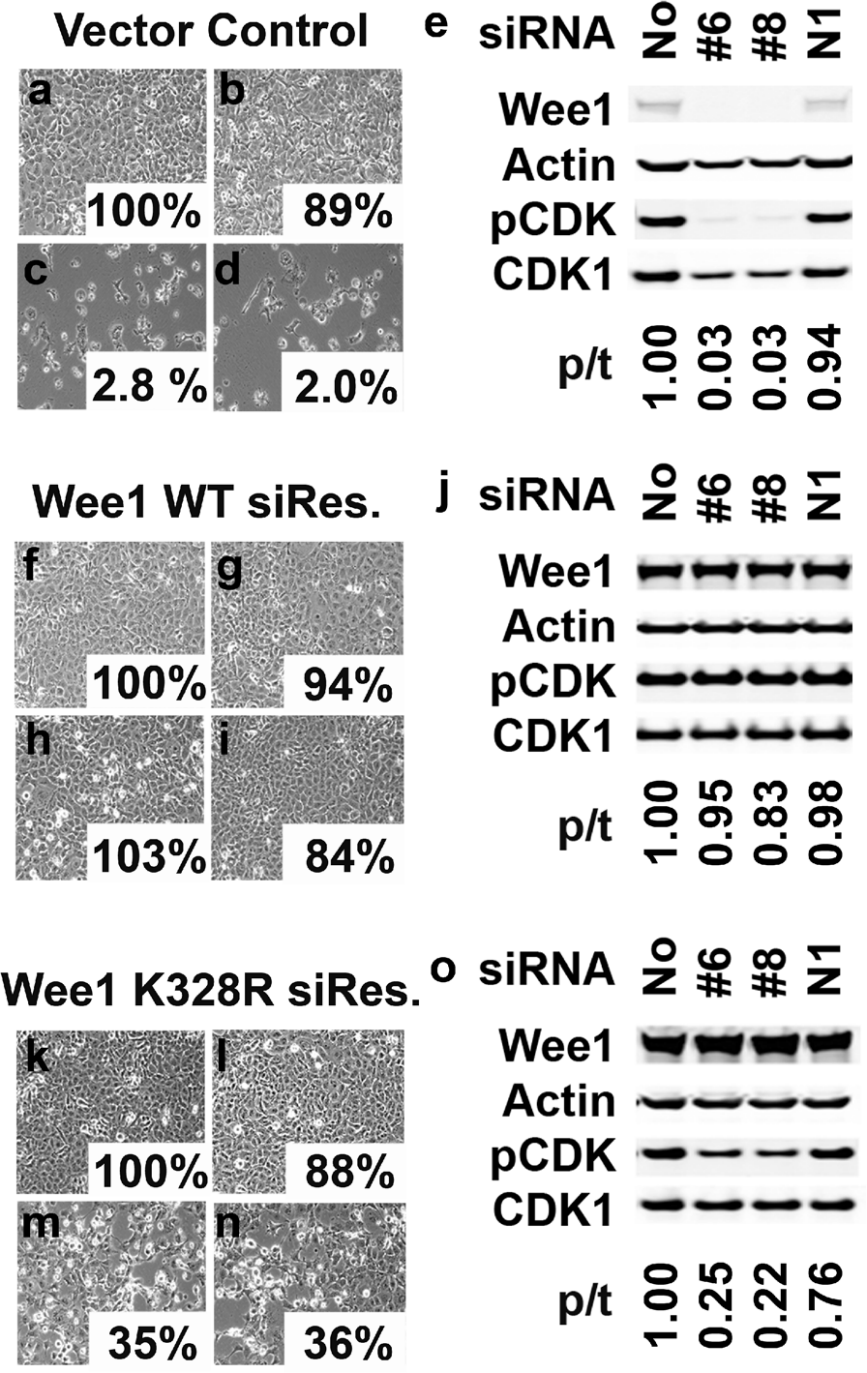
**Wee1 knockdown by siRNA treatment is specific and its effects are kinase dependent.** NCI-H1299 vector control cells were treated with no siRNA **(a)**, non-targeting siRNA (N1) **(b)**, Wee1 #6 **(c)** or Wee1 #8 **(d)** siRNAs for five days. The remaining percentage of viable cells after proliferation is given for each sample. **(e)** NCI-H1299 vector control cells were subjected to 5 nM siRNA treatment for two days following plating and analyzed by Western blot to determine effects on Wee1 knockdown and pCDK levels. The ratio of phospho/total CDK (p/t CDK) resulting from each siRNA treatment was determined by densitometry relative to their Actin controls. **(f-i)** NCI-H1299 cells over-expressing a wild type Wee1 with siRNA resistant seed regions. The samples are the same as in **a-e**. **(k-o)** NCI-H1299 cells over-expressing a kinase-altered Wee1 K328R with siRNA resistant seed regions. The samples are the same as in **a-e**. All results are representative of three separate experiments.

We performed siRNA rescue experiments to rule out the possibility of off-target effects leading to the inhibition of proliferation seen after Wee1 siRNA treatment in the subset of cell lines we assayed. To accomplish this goal, stable NCI-H1299 NSCLC cell lines with a vector control or a siRNA-resistant version of Wee1 were isolated and subjected to siRNA treatment for both proliferation and Western blot analyses. The Wee1 siRNA-resistant DNA construct had scrambled seed regions for both siRNAs #6 and #8 but still coded for the WT amino acid sequence of Wee1. Our results indicate that, while the vector control cell line responded phenotypically (proliferation) and molecularly (Western) as expected, the siRNA-resistant Wee1 cells were unaffected by treatment (Figure 3a–j). These results confirm that the siRNAs targeting Wee1 are on-target and that the dramatic cellular changes are solely a result of the loss of Wee1 protein.

Using these experimental methods we could also determine if the siRNA effects were a result of loss of a scaffolding function or kinase activity of Wee1. To answer this question, we generated a siRNA-resistant/kinase-altered version of Wee1 in which altering the critical active site lysine of Wee1 to an arginine residue (K328R) results in a decrease of Wee1 kinase activity [16]. NCI-H1299 cells over-expressing the siRNA-resistant/kinase-altered version of Wee1 were not rescued to a similar extent as the kinase-active version (Figure 3f–o). The siRNA-resistant/kinase altered Wee1 construct does produce a slight amount of phenotypic rescue (~35%) for both siRNA #6 and #8, but this can be explained by one of two likely possibilities: the K328R change may not produce a fully kinase-dead Wee1 or the over-expressed siRNA-resistant Wee1 constructs may cause some degree of siRNA protection to the endogenous Wee1 mRNA present in the cells. These effects can be seen on the molecular level when relative levels of phospho- to total-CDK (p/t CDK) are taken into account. The level of p/t CDK is essentially absent in the vector control NCI-H1299 cells (Figure 3e), restored in the siRNA-resistant cells (Figure 3j) and partial when the siRNA-resistant/kinase altered Wee1 is over-expressed (Figure 3o). These experimental results imply that the Wee1 siRNAs are on-target and that the loss of kinase activity and the subsequent downstream signaling effects are responsible for the dramatic effects of Wee1 siRNA treatment on cellular proliferation seen in a specific subset of solid tumor cell lines.

### Wee1 knockdown effects are stronger in tumor cell lines with recognized inactive p53 status

Having ruled out several plausible explanations for the drastically different phenotypic response to Wee1 siRNA treatment in our initial 5 cell lines we decided to expand the number of cell lines in our study. We added 15 more tumor cell lines for a total of 20 from various tissue origins and found that, in general, response to Wee1 siRNA treatment correlated well with inactive p53 status (Table 1). The trend in sensitivity upon Wee1 knockdown towards inactive p53 status can be observed when comparing wild-type p53 tumor cell lines (top 7 cell lines with an average 76% viability) to tumor cell lines with deletion/nonsense mutations and the E6 virus that result in cell lines with recognized inactive p53 status (bottom 6 cell lines with an average of 12.5% viability).

**Table 1:** Wee1 siRNA effects on cell line panel

**Cell line**	**Cancer origin**	**TP53 status**	**% viability**
A2780	Ovary	WT	98
A549	Lung	WT	102
D54MG	Glioma	WT	93
HCT-116	Colon	WT	74
MCF-7	Breast	WT	65
U87MG	Glioblastoma	WT	42
ZR-75-1	Breast	WT	57
U118MG	Glioblastoma	R213Q	73
HCT-15	Colon	S241F	98
DLD-1	Colon	S241F	89
DAOY	Medulloblastoma	C242F	3
MiaPaca-2	Pancreas	R248W	92
A431	Epidermis	R273H	70
MDA-MB-231	Breast	R280K	15
MDA-MB-453	Breast	368-del30	29
MDA-MB-361	Breast	S166*	5
Calu-6	Lung	R196*	7
HeLa	Cervix	Low (E6)	3
SK-N-MC	Ewing’s	-/-	27
H1299	Lung	-/-	4

Efficacy of Wee1 siRNA treatment was assayed in a panel of 20 solid human cell lines derived from various tumors. Percent viability is measured after five days by taking the average of Wee1 siRNAs #6 and #8 and comparing them to the non-targeting and Ran siRNA controls. p53 genotype data were procured from The p53 Mutation HandBook (http://p53.free.fr[17]). All results are the average of a minimum of three separate experiments.

### Direct evidence for the importance of p53 status in response to loss of Wee1 activity

**Figure 4:**
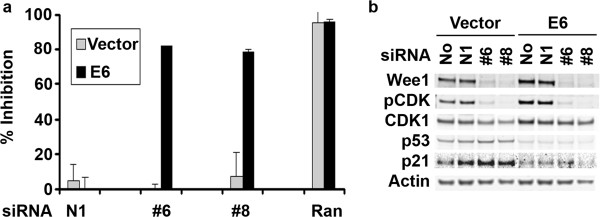
**Loss of Wee1 protein affects only the isogenic RKO inactivated p53 cell line. (a)** RKO-vector control (gray bars) and RKO-E6 (black bars) cell lines were subjected to 5 nM siRNA treatment for five days following plating. **(b)** RKO-vector control and RKO-E6 cell lines were subjected to 5 nM siRNA treatment for two days following plating. Direct cell lysates were analyzed by Western blotting to ascertain loss of Wee1 protein levels as well as the effects on its direct target (pCDK). p53 and p21 protein levels were assessed to determine effects of the HPV-E6 transgene. Total CDK1 and Actin were blotted to control for total protein levels.

The observed effects of Wee1 inhibition and their apparent relation to p53 status presented herein and elsewhere [8,12] suggested that abrogation of p53 in a normally p53 wild-type setting would generate sensitivity to Wee1 inhibition. To directly test this hypothesis we performed experiments in isogenic pairs of the colorectal carcinoma cell line RKO over-expressing either a vector control or the human papillomavirus protein E6. E6 expression is associated with a substantial decrease in p53 protein levels in these cells resulting in a clonal cell line with inactive p53 [18]. We performed siRNA experiments and found dramatic inhibition of proliferation in the RKO-E6 cells treated with Wee1 siRNAs while the RKO-vector cells were unperturbed (Figure 4a). Western blot analyses revealed that Wee1 protein knockdown and subsequent loss of cellular activity as shown by pCDK levels were roughly equivalent in both cell lines although there was a marked difference in the levels of p53 protein and its transcriptional target p21 in the two cell lines (Figure 4b).

### Loss of Wee1 activity results in caspase-dependent apoptosis in p53-null cells

**Figure 5:**
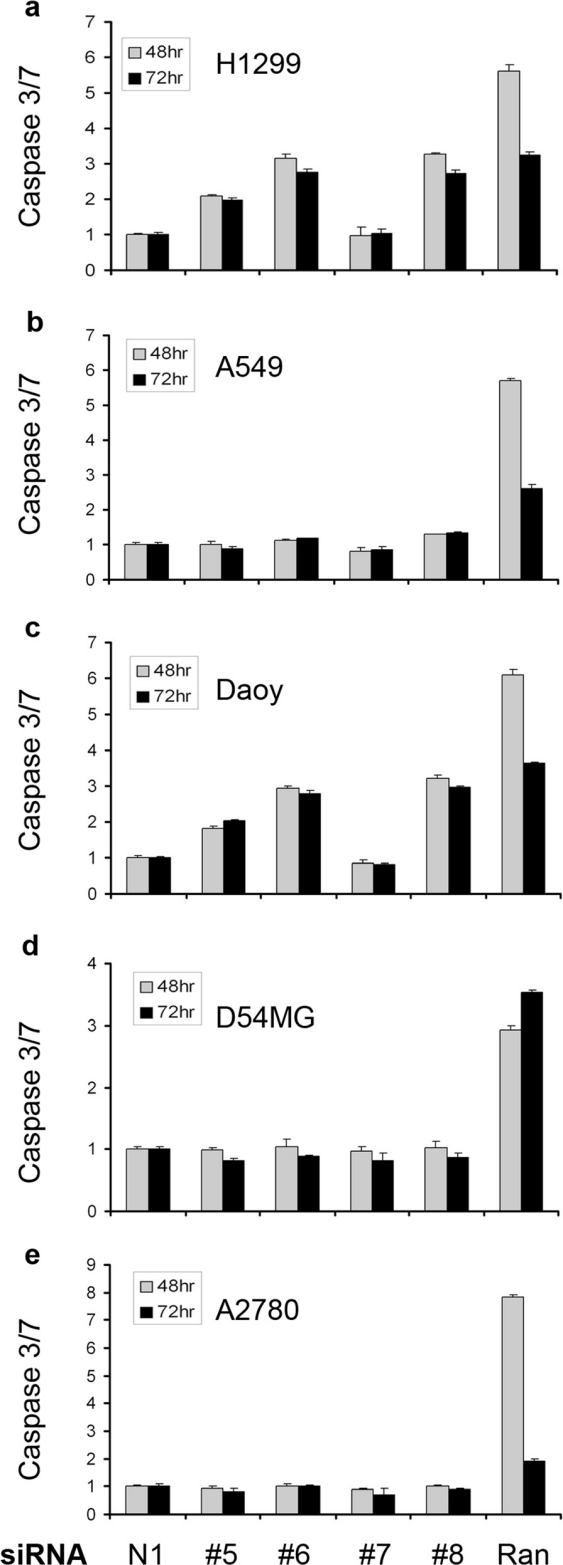
**Wee1 knockdown results in caspase-dependent apoptosis in a subset of solid tumor cell lines. (a)** NCI-H1299, **(b)** A549, **(c)** Daoy, **(d)** D54MG and **(e)** A2780 cells were subjected to siRNA treatment for 48 (gray bars) or 72 hours (black bars) and assayed for induction of Caspase-3/7 using the Caspase-Glo 3/7 assay. Results shown indicate fold induction of Caspase-3/7 activity.

In an attempt to decipher the potential mechanisms by which Wee1 ablation affects cell growth, we performed caspase-activity assays after Wee1 siRNA treatment. Wee1 siRNAs resulted in an induction of Caspase 3/7 activity in a time-dependent fashion in NCI-H1299 and Daoy cells but did not affect A549, D54MG and A2780 cells (Figure 5). These effects correspond well to the cellular proliferation data seen in Figure 1 and suggest that these cells are dying from caspase-dependent apoptosis rather than inhibition of proliferation from cytostatic effects. This caspase-dependent apoptosis is rescued in cells over-expressing the siRNA-resistant Wee1 construct (data not shown). Additionally, the likely cell-cycle related effects of Wee1 knockdown resulted in activation of caspase activity at a slower rate than the positive control Ran siRNA. For example, when comparing the Caspase-3/7 activity at 48 and 72 hours it should be noted that effects of Ran siRNA in cells peaks at 48 hours and decreases in most cell lines by 72 hours, presumably because most cells have already undergone apoptosis. However, Wee1 knockdown has a slower rate of caspase induction that begins at ~ 48 hours and remains steady at 72 hours (Figure 5 and data not shown).

### Loss of Wee1 activity has dramatic cell cycle effects in a subset of tumor cell lines

**Figure 6:**
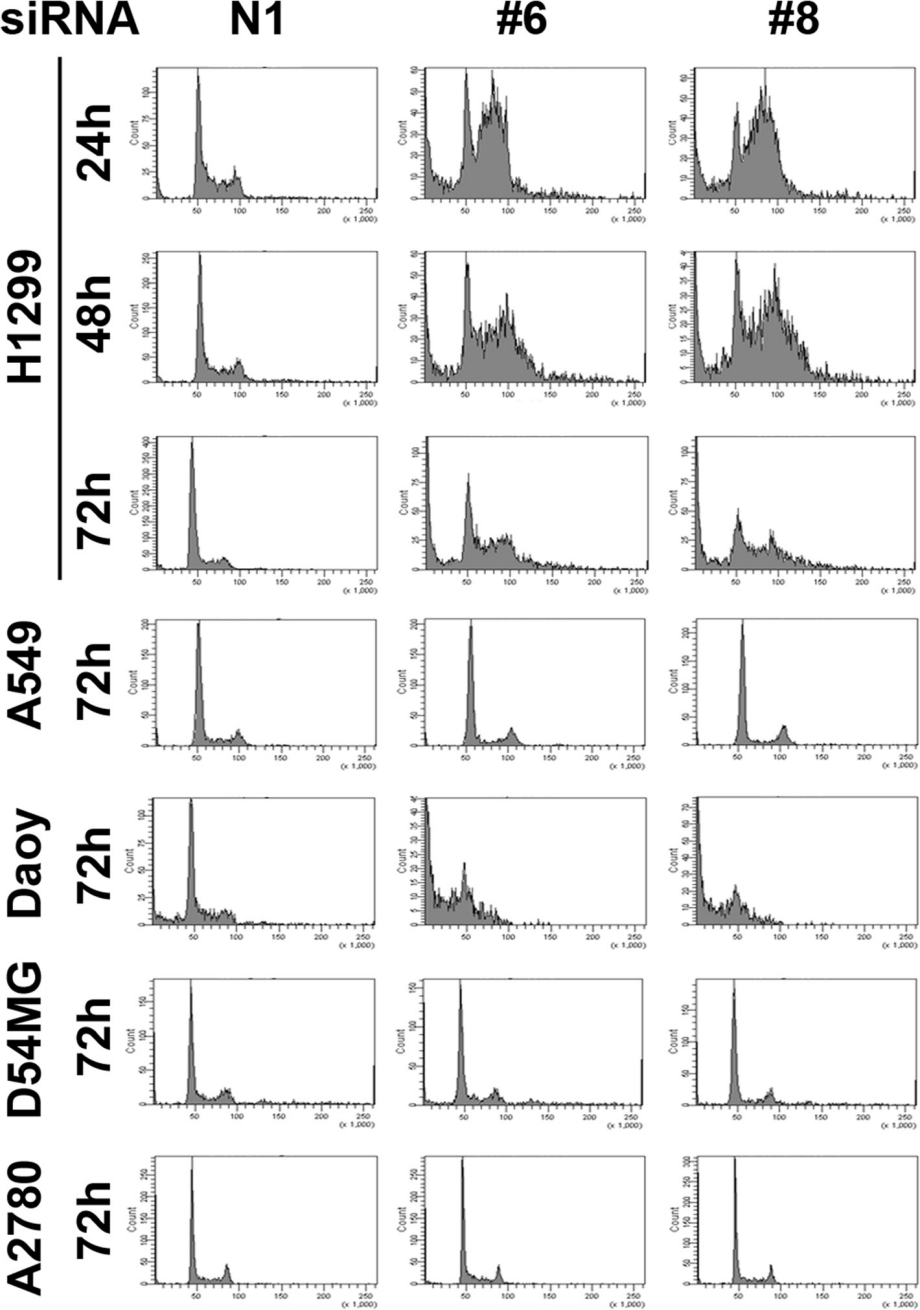
**Wee1 knockdown causes dramatic cell cycle effects cell cycle in a subset of solid tumor lines.** Cells were subjected to 5nM non-targeting (N1) or Wee1 siRNAs (#6 and #8) and stained with PI for FACS analysis. NCI-H1299 cells were analyzed for DNA content at 24, 48 and 72 hours after siRNA transfection. DNA content of A549, Daoy, D54MG and A2780 cells were analyzed at 72 hours after siRNA transfection.

The timing of caspase activity induction and the known functions of Wee1 at both the mitotic and DNA synthesis checkpoints suggested that cell death in sensitive lines was related to cell cycle control. To interrogate this possibility, we performed propidium iodide (PI) DNA staining of cells and performed flow cytometry at time points after Wee1 siRNA treatment. A549, D54MG and A2780 cells had unperturbed cell cycles after Wee1 knockdown that were indistinguishable from a non-targeting siRNA control at 24, 48 and 72 hours after treatment (Figure 6 and data not shown). However, two cell lines with impaired p53 activity, NCI-H1299 and Daoy, had significant cell cycle effects after Wee1 knockdown as compared to the non-targeting siRNA control at all time points analyzed (Figure 6 and data not shown). Loss of Wee1 kinase activity in NCI-H1299 cells resulted in a substantial accumulation of DNA content greater than 2 N that corresponded to a mixture of S-phase, G2/M and >4 N DNA content in cells that ultimately led to a significant fraction of sub-G1 DNA content corresponding with cell death that peaks at 48 hours post-transfection (Figure 6). We also visualized NCI-H1299 and A549 cells by live cell imaging after transfection with Wee1 and controls siRNAs (Additional files 4, 5, 6 and 7) and found that the A549 cells treated with control and Wee1 siRNAs were indistinguishable in phenotype and growth rates. However, NCI-H1299 cells treated with Wee1 siRNA appear to start and abort mitosis at more rapid time periods than their respective non-targeting siRNA control counterparts as visualized by cellular shape changes from flat to round [19]. The cumulative results shown in Figure 6 and the time-lapse light microscopy movies suggest that NCI-H1299 and Daoy cellular DNA content may be undergoing concomitant attempts to perform DNA synthesis and mitosis leading to aneuploidy and incomplete DNA synthesis and this ultimately leads to apoptotic cell death after treatment with Wee1 siRNA.

### Loss of Wee1 activity results in mistimed cell cycle events

**Figure 7:**
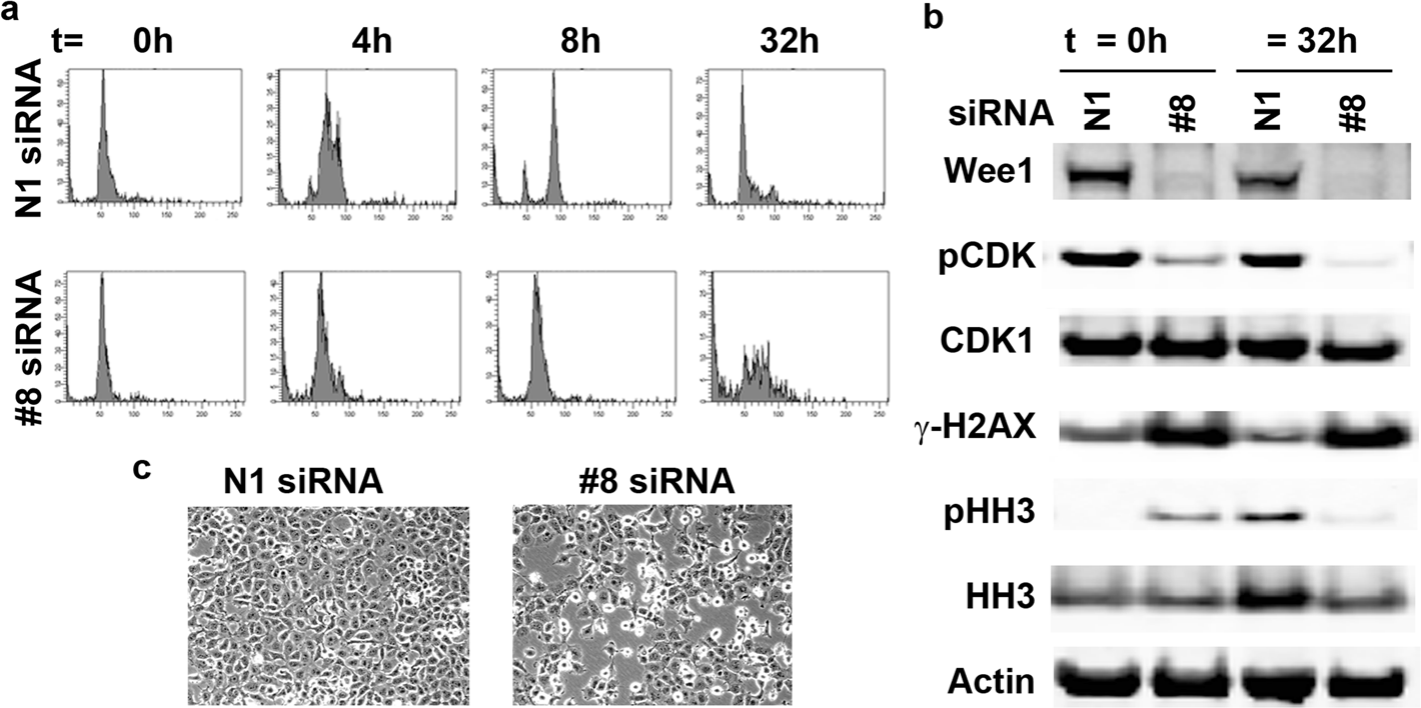
**Wee1 siRNA treatment results in mistimed cell cycle events in NCI-H1299 cells. (a)** NCI-H1299 cells were synchronized in S-phase using double thymidine block and treated with non-targeting (N1) or Wee1 (#8) siRNAs. DNA content was measured by FACS analysis of PI staining of cells at 0, 4, 8 and 24 hours after release. **(b)** NCI-H1299 cells were treated as in **(a)** and directly lysed and analyzed by Western blotting at 0 and 32 hours after release. **(c)** NCI-H1299 cells were treated as in **(a)** and representative light microscopy phenotypic images were captured 24 hours after release.

Cell populations can be synchronized at specific stages of the cell cycle through various means including serum starvation and chemical methods. However, several of these methods are harsh and not amenable to co-treatment with siRNA because of the length of time required for turnover of endogenous protein levels to decrease. We found that Wee1 siRNA treatment resulted in optimal loss of Wee1 protein at 16 hours post-transfection (data not shown). With this timing limitation in mind, we decided upon double thymidine block to synchronize cells in late G1/early S-phase because of its relative lack of toxicity coupled with the length of treatment. NCI-H1299 cells treated with non-targeting or Wee1 siRNAs were also subjected to synchronization and analyzed for DNA content and Western blot at time points following thymidine block release. The cells treated with control siRNA proceeded through the cell cycle from the S-phase block to G2/M and back to G1 as expected, but the cells without Wee1 activity never make it past S-phase and eventually die (Figure 7a). Of particular interest is that cells treated with Wee1 siRNA already have the mitotic marker phospho-Histone H3 (pHH3) present at the release point (t = 0 h) as seen by Western blot analysis (Figure 7b). This demonstrates that despite the lack of G2 DNA content in these cells that they still enter M-phase. This results in DNA damage that is again visible at the release from thymidine block in Wee1 knockdown cells as seen by the increased signal of the rapid response DNA damage marker γ-H2AX (Figure 7b). The gross phenotype of NCI-H1299 cells without functional Wee1 at 24 hours after thymidine release also suggests an abnormal percentage of cells in a “rounded” mitotic state (Figure 7c).

### Wee1 knockdown results in incorrect timing of DNA replication origin firing

**Figure 8:**
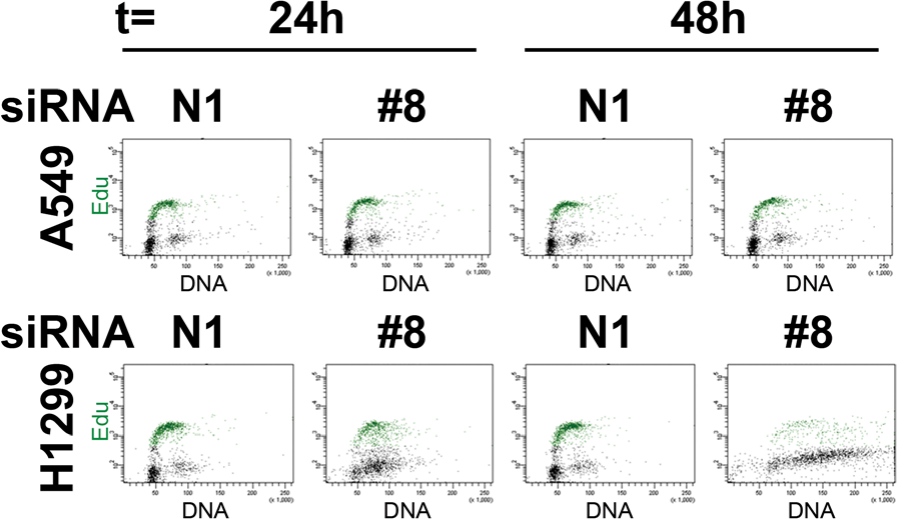
**Loss of Wee1 activity results in incorrect initiation of DNA synthesis in NCI-H1299 cells but not A549 cells.** NCI-H1299 and A549 cells were treated with 5 nM non-targeting (N1) or Wee1 (#8) siRNAs and stained for DNA content (X-axis) and EdU incorporation (Y-axis) to mark cells in S-phase. Samples were taken at both 24 and 48 hours after treatment.

To look more closely at the non-mitotic effects resulting from the loss of Wee1 kinase activity, we performed EdU incorporation assays in both A549 and NCI-H1299 cells. We transfected Wee1 siRNA and a non-targeting siRNA control and performed dual Edu and DNA content flow cytometry on cells at 24 and 48 hours after treatment (Figure 8). In this assay, A549 cells treated with either siRNA had G1 DNA content that could incorporate EdU until a DNA content of 4 N is reached corresponding to the G2/M stage of the cell cycle indicating the lack of premature mitosis in these cells. NCI-H1299 cells with Wee1 knocked down continued to accumulate DNA content (>4 N) while still trying to add more DNA as seen by incorporation of EdU (Figure 8). These results are reflective of the recently reported effects of Wee1 siRNA treatment in U2OS cells resulting in increased activity of both CDK1 and CDK2 kinase activity [14].

## Discussion

Wee1 is a master regulator of cellular integrity and its activity keeps normal cells from proceeding through the S-phase and G2/M checkpoints before their genomic content is adequately prepared to do so [6,7]. We found Wee1 to be over-expressed in a number of solid primary tumors (data not shown, http://htp://www.oncomine.com) and this has recently been demonstrated in primary glioblastomas [10]. These data suggest that Wee1 may play a role in some cancer cells to maintain a level of genetic stability to enable the growth advantage conferred by their new cancer karyotype. We hypothesized that cancer cells would be susceptible to loss of Wee1 kinase activity based on the premise that all metazoan cells, including those in neoplasms, rely on the correct timing of cell cycle events for their survival and proliferation. Our studies indicate that this hypothesis is correct in a subset of solid tumor cell lines with inactive p53. Upon loss of Wee1 kinase activity resulting from specific siRNA knockdown, these cells undergo DNA synthesis while attempting simultaneous mitosis, ultimately leading to mitotic catastrophe and caspase-dependent apoptosis.

p53 is a short-lived transcription factor that acts as a tumor suppressor to detect and eliminate incipient cancerous cells. p53 activity results from a complex network involving the DNA damage response, transcriptional activation and post-translational modifications and results in regulation of hundreds of genes [20]. Neutralization of p53 function is a major hallmark of tumor cells and this loss can be the result of direct somatic mutation, deletion, proteasomal degradation or sequestration to achieve a pathologic survival advantage [21]. One such protein that mediates degradation of p53 that we have utilized in this study is the human papillomavirus protein E6. In addition to the numerous methods to hinder p53 activity mentioned above, analyzing p53 function in cells is further complicated by the genetic redundancy inherent to cells in the form of the other members of the p53 gene family, TP63 and TP73 [22]. As a result, loss of p53 activity is difficult to categorize by simple direct genotyping methods of TP53. This can be best witnessed within our cell line panel data in response to Wee1 knockdown. While there certainly appears to be a trend towards TP53 status, there are exceptions to this rule including the U87MG and MCF-7 WT p53 cell lines. To attempt to avoid some of the inherent complexities of the p53 tumor suppressor pathway, we focused our study on cell lines that have well characterized p53 activity in the literature, including the p53 null NCI-H1299 and p53 WT A549 cell lines, as well as the isogenic RKO pair of cell lines. Therefore, we can only conclude from our studies that, in general, there is a susceptibility of p53 inactive cells to respond to Wee1 knockdown.

Previous studies have linked p53 status to cellular sensitivity to loss of Wee1 by siRNA or small molecule inhibitors but always in the presence of cytotoxic agents [8,10-12,14]. The proposed hypothesis for this mechanism is that cells with inactive p53 have a defective G1/S checkpoint and therefore can be sensitized to inhibition of the G2/M checkpoint in combination with DNA-damaging agents [23]. However, our results demonstrate that specific loss of Wee1 kinase activity results in inhibition of proliferation and apoptosis in a subset of solid tumor cell lines in the absence of co-treatment with cytotoxic agents. Therefore, a highly specific small molecule inhibitor targeting only the cell cycle suppressor Wee1 should kill p53 inactive tumor cells in the absence of cytotoxic treatment. It should be noted that, while p53 status is difficult to ascertain due to its complexity, most normal tissues would have a functioning p53 tumor suppressor pathway.

Our findings reveal that Wee1 inhibition affects both the G1/S and G2/M transitions through the cell cycle. Wee1 is capable of inactivating both the CDK2-CyclinE and CDK2-CyclinB complexes [6,7] and the Y15 specific antibody used in this study and others is identical in both CDK1 and CDK2. Therefore, loss of Y15 phosphorylation seen after Wee1 siRNA knockdown may correspond to either or both of these cyclin dependent kinases. We have presented data consistent with the hallmarks of activation of both of these kinases including phospho-Histone H3 upregulation (CDK1) and DNA synthesis marked both by DNA content > 4 N as well as continued incorporation of EdU into cells with higher DNA content (CDK2). It should be noted that we did not observe similar effects upon siRNA knockdown of the other members of the DNA damage response including CHK1/2 and the related CDK regulatory threonine kinase Myt1 (data not shown). It appears therefore that Wee1 acts as the major regulatory kinase in the control of the G1/S and G2/M checkpoints. This is likely because of its direct proximity in the DNA damage response to the CDKs and the importance of the Y15 site over the T14 site phosphorylated by Myt1.

## Conclusions

In this study we demonstrate that loss of Wee1 protein results in dramatic cell cycle events, including mitotic catastrophe and mistimed DNA synthesis, ultimately resulting in caspase-dependent apoptosis in a subset of p53 inactive cells. We have achieved this by using siRNA knockdown and provided evidence that this knockdown is specific and kinase dependent through siRNA rescue studies. One compelling finding of this study that will require further analyses is how the subset of p53 active cells are able to accommodate the loss of Wee1 activity and proceed through the cell cycle and proliferate at an equivalent rate to the no siRNA and non-targeting siRNA controls. We postulate loss of Wee1 results in activation of the DNA damage responses and that p53 inactive cells cannot rebalance CDK activities and ultimately have cell cycle mistiming and die via apoptosis. In conclusion, we believe that these data have important ramifications for the treatment of malignancies, and a renewed effort to identify highly specific small molecule inhibitor of Wee1 could provide an unmet clinical need in the treatment of solid tumors.

## Abbreviations

CDK: Cyclin-dependent kinases; siRNA: Small interfering RNA; NSCLC: Non-small cell lung; pHH3: Phospho-Histone H3.

## Competing interests

All authors are employees of AbbVie (North Chicago, IL, U.S.A.). All research conducted herein was funded by AbbVie. AbbVie contributed to the study design, research and interpretation of data, writing, reviewing and approving the manuscript.

## Authors’ contributions

WP conceived the study, performed experiments, participated in generating figures and drafted the manuscript. QZ performed experiments and participated in generating figures. LT performed experiments. CT participated in the design of the study. JW participated in the design and coordination of the study. All authors read and approved the final manuscript.

## Pre-publication history

The pre-publication history for this paper can be accessed here:

http://www.biomedcentral.com/1471-2407/14/430/prepub

## Supplementary Material

Additional file 1 **Proliferation results upon Wee1 knockdown are similar using both metabolic and DNA content readouts.** NCI-H1299 **(a)** and A549 **(b)** cells treated with non-targeting (N1), Wee1 (#8) and Ran siRNAs at both 5 and 20 nM for five days measured for viability using MTS reagent. NCI-H1299 **(c)** and A549 **(d)** cells treated with non-targeting (N1), Wee1 (#8) and Ran siRNAs at both 5 and 20 nM for five days measured for DNA content using CyQuant reagent. Click here for file

Additional file 2 **Wee1 siRNA treatment produces efficient target knockdown in A2780 cells.** A2780 cells were subjected to 5 nM siRNA treatment for two days following plating. Non-targeting siRNA (N1) and Wee1 siRNAs #5-#8 were all analyzed. Direct cell lysates were analyzed by Western blotting to ascertain loss of Wee1 protein levels as well as the effects on its direct target (pCDK). Total CDK1 and Actin were blotted to control for total protein levels. Click here for file

Additional file 3 **Wee1 siRNA treatment is effective over a wide-range of concentrations.** Daoy cells were subjected to 0.2, 1, 5 and 20 nM siRNA treatment for two days following plating. Direct cell lysates were analyzed by Western blotting to ascertain loss of Wee1 protein levels as well as the effects on its direct target (pCDK). Total CDK1 and Actin were blotted to control for total protein levels. Click here for file

Additional file 4 NCI-H1299 cells transfected with control (NT1) siRNA. Click here for file

Additional file 5 NCI-H1299 cells transfected with Wee1 (#8) siRNA. Click here for file

Additional file 6 A549 cells transfected with control (NT1) siRNA. Click here for file

Additional file 7 A549 cells transfected with Wee1 (#8) siRNA. Click here for file
