# Supplementary figures and images for: Genetic inhibition of the atypical kinase Wee1 selectively drives apoptosis of p53 inactive tumor cells
Source: BMC Cancer. 2014 Jun 13;14:430. doi: 10.1186/1471-2407-14-430 (PMC4229861; doi:10.1186/1471-2407-14-430)

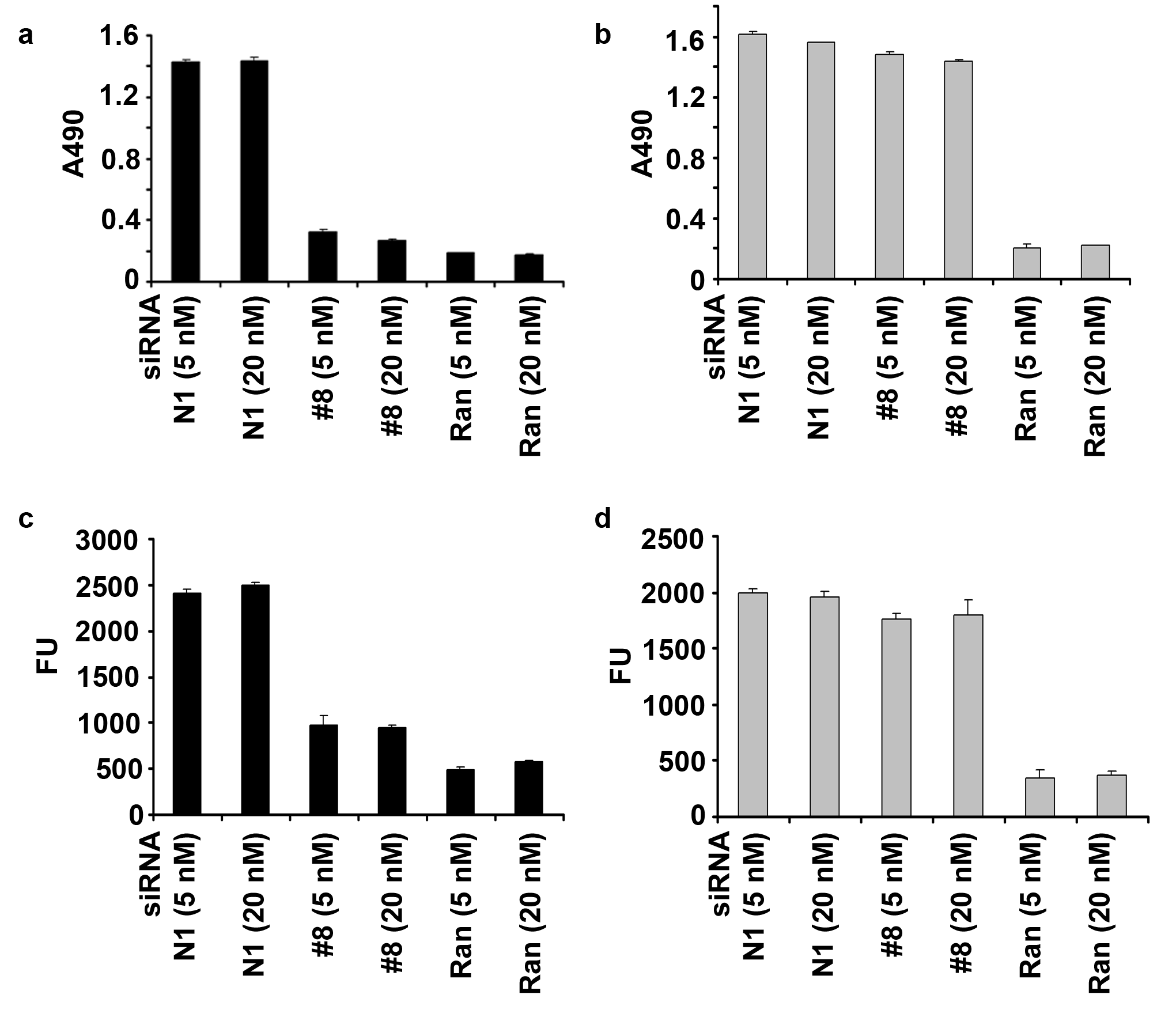

Supplement: Additional file 1 — Proliferation results upon Wee1 knockdown are similar using both metabolic and DNA content readouts. NCI-H1299 (a) and A549 (b) cells treated with non-targeting (N1), Wee1 (#8) and Ran siRNAs at both 5 and 20 nM for five days measured for viability using MTS reagent. NCI-H1299 (c) and A549 (d) cells treated with non-targeting (N1), Wee1 (#8) and Ran siRNAs at both 5 and 20 nM for five days measured for DNA content using CyQuant reagent. [file 1471-2407-14-430-S1.tiff]

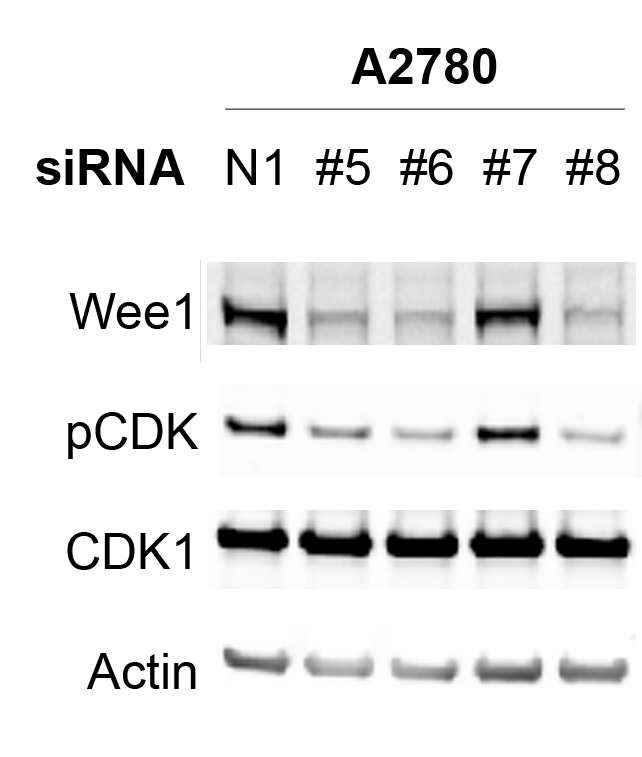

Supplement: Additional file 2 — Wee1 siRNA treatment produces efficient target knockdown in A2780 cells. A2780 cells were subjected to 5 nM siRNA treatment for two days following plating. Non-targeting siRNA (N1) and Wee1 siRNAs #5-#8 were all analyzed. Direct cell lysates were analyzed by Western blotting to ascertain loss of Wee1 protein levels as well as the effects on its direct target (pCDK). Total CDK1 and Actin were blotted to control for total protein levels. [file 1471-2407-14-430-S2.tiff]

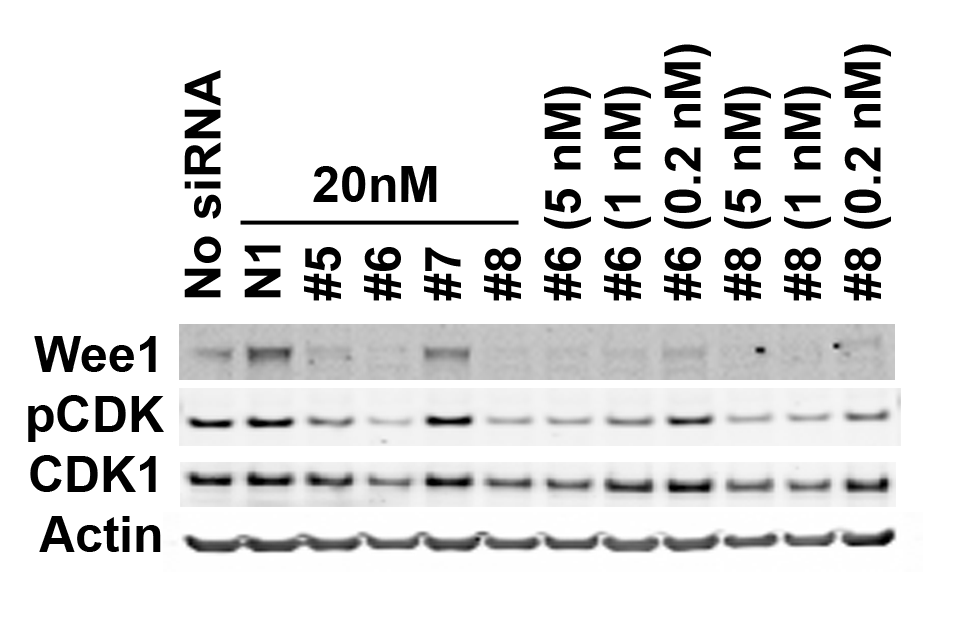

Supplement: Additional file 3 — Wee1 siRNA treatment is effective over a wide-range of concentrations. Daoy cells were subjected to 0.2, 1, 5 and 20 nM siRNA treatment for two days following plating. Direct cell lysates were analyzed by Western blotting to ascertain loss of Wee1 protein levels as well as the effects on its direct target (pCDK). Total CDK1 and Actin were blotted to control for total protein levels. [file 1471-2407-14-430-S3.tiff]
